# Current practice and surgical outcomes of neoadjuvant chemotherapy for early breast cancer: UK NeST study

**DOI:** 10.1093/bjs/znac131

**Published:** 2022-05-11

**Authors:** Hiba Fatayer, Rachel L O’Connell, Finian Bannon, Charlotte E Coles, Ellen Copson, Ramsey I Cutress, Rajiv V Dave, Matthew D Gardiner, Margaret Grayson, Christopher Holcombe, Sheeba Irshad, Gareth W Irwin, Ciara O’Brien, Carlo Palmieri, Abeer M Shaaban, Nisha Sharma, Jagdeep K Singh, Ian Whitehead, Shelley Potter, Stuart A McIntosh, NeST Study Research Collaborative, H Curry, E Iddles, M Mahmood, Y Masannat, J Schneider, L Simpson, M Sidapra, L Baker, H Capitelli-McMahon, M Hughes, A Isaac, B Skelly, C Sirianni, N Hirst, R Linforth, A Botes, T Robinson, T Schrire, J Alfred, H Lennon, D Dumitru, E Kleidi, F Hoar, E MacInnes, K Sharma, T Alaguthurai, N Chand, C A Farulla, A Hayward, B Pearce, M Tatterton, S Laws, J Iqbal, M S Mirza, K V Sainarayanan, L Humphreys, S Tayeh, S Jones, A Ansari, R Bate, B C J Wei, B Gurung, F M T Leone, C Mitchell, G Mondani, S Pilgrim, T Sun, G Boundouki, R Broadbent, A Khan, F Morgans-Slader, J Rai, R Soulsby, H Cain, R Thomas, B Elsberger, G Walls, S Cadwell-Sneath, J Couch, M D’Auria, C Grundy, S Hitchin, H Khout, F Latief, J Mondani, A Nessa, G Oni, L Sawers, S S Rajan, Q Tan, L Whisker, A Ghoneima, M Rezacova, N Marikakis, L Ballance, U Andaleeb, N Basu, T Hubbard, A Maxwell, M Roland, C Weerasinghe, Q Ain, G Bitsakou, C Chamberlain, N Chopra, A Micha, C Norman, P Padmanabhan, N Patani, K Shanthakunalan, E St John, S Jafferbhoy, C Bransgrove, A Hussein, J Livingstone, O Waker, J Hack, S Hadad, J Newell, A Heetun, A Hargreaves, E Rahman, R Vidya

**Affiliations:** Liverpool Breast Unit, Liverpool University Hospitals NHS Foundation Trust, Liverpool, UK; Department of Breast Surgery, Royal Marsden NHS Foundation Trust, Sutton, UK; Centre for Public Health, Queen’s University Belfast, Institute of Clinical Science, Belfast, UK; Department of Oncology, University of Cambridge, Cambridge, UK; Cancer Sciences Academic Unit, Faculty of Medicine, University of Southampton and University Hospital Southampton, Southampton, UK; Cancer Sciences Academic Unit, Faculty of Medicine, University of Southampton and University Hospital Southampton, Southampton, UK; Nightingale Breast Cancer Centre, Wythenshawe Hospital, Manchester University NHS Foundation Trust, Manchester, UK; Department of Plastic Surgery, Wexham Park Hospital, Frimley Health NHS Foundation Trust, Slough, UK; Kennedy Institute of Rheumatology, Nuffield Department of Orthopaedics, Rheumatology and Musculoskeletal Sciences, University of Oxford, Oxford, UK; Northern Ireland Cancer Research Consumer Forum, Northern Ireland Cancer Trials Network, Belfast City Hospital, Belfast, UK; Liverpool Breast Unit, Liverpool University Hospitals NHS Foundation Trust, Liverpool, UK; Guy’s Cancer Centre, Guy’s and St Thomas’ NHS Trust, London, UK; School of Cancer and Pharmaceutical Sciences, King’s College London, London, UK; Breast Surgery Department, Belfast City Hospital, Belfast Health and Social Care Trust, Belfast, UK; Department of Medical Oncology, Christie Hospital NHS Foundation Trust, Manchester, UK; School of Medical Sciences Faculty of Biology, Medicine and Health University of Manchester, Manchester, UK; University of Liverpool, Institute of Systems, Molecular and Integrative Biology, Department of Molecular and Clinical Cancer Medicine, Liverpool, UK; Clatterbridge Cancer Centre NHS Foundation Trust, Liverpool, UK; Department of Pathology, Queen Elizabeth Hospital Birmingham and University of Birmingham, Birmingham, UK; Breast Unit, St James’s Hospital, Leeds, UK; Surrey and Sussex Healthcare NHS Trust, East Surrey Hospital, Redhill, UK; Liverpool Breast Unit, Liverpool University Hospitals NHS Foundation Trust, Liverpool, UK; Bristol Centre for Surgical Research, Population Health Sciences, Bristol Medical School, Bristol, UK; Bristol Breast Care Centre, North Bristol NHS Trust, Southmead Hospital, Bristol, UK; Patrick G Johnston Centre for Cancer Research, Queen’s University Belfast, Belfast, UK

## Introduction

Neoadjuvant chemotherapy (NACT) is increasingly being used to treat early breast cancer, and offers several advantages, including reducing the extent of breast and axillary surgery, and providing an *in vivo* assessment of tumour sensitivity to treatment^1–4^. Clinical trials have identified tumour subgroups with high rates of pCR. A pCR can be achieved in 45–90 per cent of human epidermal growth factor receptor 2-positive (HER2+) tumours and triple-negative breast cancer (TNBC), but the rate in oestrogen receptor-positive (ER+)/HER2-negative (HER2–) breast cancer remains below 10 per cent^3,5^. Historically, increasing pCR rates following NACT have not translated into more breast-conserving surgery (BCS), but more recent data suggest that NACT can result in surgical downstaging^6^.

NACT use in the UK appears inconsistent, as highlighted by a recent prospective audit^7^. Although UK guidelines suggest considering NACT in patients with HER2+ cancers and TNBC, detailed guidance does not exist^8^. Furthermore, it is unclear whether pCR rates in routine clinical practice reflect those observed in trials, and whether tumour downstaging influences surgical decision-making beyond the trial setting. Moreover, there remains a lack of consensus on whether definitive surgery should aim to excise the original or post-treatment tumour footprint, as highlighted in a recent UK survey^9^, in which 24 per cent of centres stated that their routine practice was to excise the original tumour footprint rather than carry out response-adapted surgery.

This prospective study aimed to determine surgical decision-making in the breast and axilla and pCR rates in routine clinical practice following NACT for early breast cancer.

## Methods

The NeST study protocol has been published previously^10^. Briefly, this was a prospective multicentre cohort study, including consecutive patients in participating units undergoing NACT as primary treatment for breast cancer, between 1 December 2017 and 30 November 2018. Demographics, multidisciplinary team (MDT) recommendations for NACT and preoperative planning, operative outcomes, and oncological data were collected for each participant. MDTs were asked to record prospectively whether patients were eligible for breast conservation at diagnosis. A pCR was defined by the absence of residual invasive disease with or without the presence of residual *in situ* disease (ypT0/ypTis) with negative axillary nodes (ypN0)^11^. Further details are provided in the *Supplementary material*.

## Results

A total of 1283 patients were entered into the NeST study database from 39 UK units; complete histopathological and surgical outcomes data were available for 900 patients (916 tumours). Patient demographics and baseline tumour characteristics are summarized in *Table S1*.

### Pathological response

A pCR in the breast (ypT0/ypTis) was reported in 379 tumours (41.4 per cent), and 330 of 448 (36.0 per cent) node-positive tumours (ypT0/is, ypN0). The pCR rates by tumour subtype are summarized in *Table 1* and pCR rates in patients with node-positive disease by subtype in *Table S2*.

**Table 1:** Overall pathological response rates by tumour subtype in both the breast and breast/axilla

		Final pathological response				
		pCR		pPR	No response	Total
**Pretreatment pathology**	ypT0	ypT0/ypTis	ypT0/yTis, yN0			
**HER2+**	166 (38.9)	236* (55.1)	205* (48.1)	180* (42.3)	10 (2.3)	426 (46.5)
HER2+/ER+	87 (31.9)	129 (47.3)	115 (42.1)	137 (50.2)	7 (2.7)	273 (29.8)
HR2+/ER–	79 (52.3)	106 (70.2)	85 (56.3)	42 (27.8)	3 (2.0)	151 (16.5)
**TNBC**	89 (33.9)	110 (41.9)	101 (38.5)	129 (49.2)	23 (8.8)	262 (28.6)
**ER+/HER2–**	27 (11.8)	34 (14.9)	24 (10.5)	175 (76.8)	19 (8.3)	228 (24.9)
**Total**	282 (30.8)	379 (41.4)	330 (36.1)	484 (52.9)	52 (6.1)	916

Values in parentheses are percentages. A pCR was defined by the absence of residual invasive disease (ypT0) but *in situ* disease could be present (ypTis); absence of residual disease in the breast or axilla was classified as ypT0/ypTis, ypN0. Patients with unknown oestrogen receptor (ER) status on core biopsy were excluded. pPR, pathological partial response; HER2, human epidermal growth factor receptor 2; TNBC, triple-negative breast cancer.

### Surgical management

**Fig. 1:**
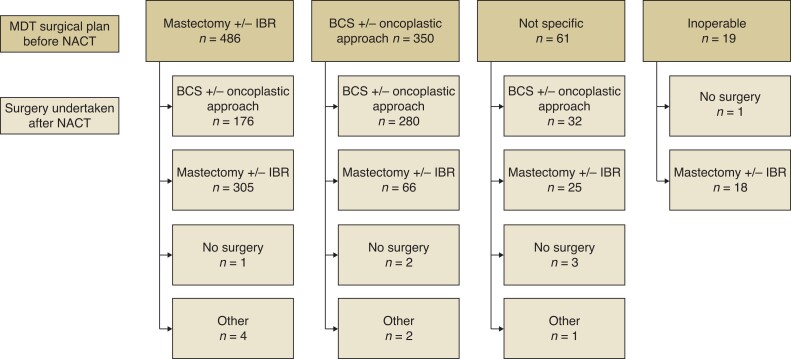
Surgical management, showing surgical plan at diagnosis and actual surgical management after systemic therapy MDT, multidisciplinary team; NACT, neoadjuvant chemotherapy; IBR, immediate breast reconstruction; BCS, breast-conserving surgery.

*Figure 1* shows the initial surgical management plan and actual treatment received. At diagnosis, 486 tumours were ineligible for BCS, and were 350 deemed suitable for BCS. Following NACT, operation for 176 of 486 tumours (36.2 per cent) was converted from mastectomy to BCS. Downstaging rates varied according to subtype; 48.8 per cent of TNBC, 32 per cent of HER2+, and 30 per cent of ER+/HER2– procedures were converted to BCS (*P* = 0.004; χ^2^ test). Among 350 patients suitable for BCS at diagnosis, 280 underwent BCS and 66 had a mastectomy. A breast pCR was reported in 143 patients (34.5 per cent) who had a mastectomy and 231 (47.3 per cent) who underwent BCS (*P* < 0.001; Fisher’s exact test). Of 176 tumours downstaged from mastectomy, 45 per cent had a pCR and 50 per cent a partial response to NACT.

The intention to resect either the original breast tumour or post-treatment tumour footprint was stated for 855 patients: resection of the original tumour footprint in 383 (44.8 per cent) and of the post-treatment tumour footprint in 472 (55.2 per cent). After BCS, 54 patients (11.4 per cent) had involved margins. Of these, surgery in 22 (40.7 per cent) was downstaged to BCS from an original plan for mastectomy, and 28 (51.9 per cent) were eligible for BCS at baseline. Further surgery was recommended for 40 patients, BCS in 34 (85 per cent) and completion mastectomy in 6 (15 per cent). The final overall mastectomy rate was 45.9 per cent when re-excision was considered.

### Axillary surgery

Nodal status at diagnosis is shown in *Table S1*. Sentinel lymph node biopsy (SLNB) was planned before NACT in 42 patients (9.4 per cent) with cN0 disease, and after NACT in 372 (82.9 per cent). Among cN1+ axillae, radiological reassessment after NACT was planned in 149 (32.3 per cent) and carried out in 112, with disease in 70 patients (62.5 per cent) downstaged to cN0. The axillary surgery performed was recorded for 908 of 916 tumours (99.1 per cent) (*Table S3*). SLNB was undertaken after NACT in 405 (44.6 per cent), with pN+ reported on final histology in 30 patients (7.4 per cent). Targeted axillary dissection was carried out in 55 (6 per cent), and the majority had pN0 disease (85.5 per cent). In total, 385 patients had axillary lymph node dissection after NACT and, of these, 186 (48.3 per cent) had pathologically negative nodes.

## Discussion

This prospective multicentre cohort study reports real-world pCR rates after NACT, which are broadly comparable to those in clinical trials, with pCR rates of 41.9 per cent (TNBC), 47.3 per cent (HER2+/ER+), and 70.3 per cent (HER2+/ER-negative), in keeping with published data. With respect to ER+/HER2– breast cancer, it is known that pCR rates in such tumours are low, the rate of 14.9 per cent in the present series being in line with reported rates of 7.5–15.2 per cent. Despite this, NACT remains commonly used in ER+/HER2– disease, as indicated by this group making up 24.9 per cent of patients in the present study. This finding highlights the need for careful consideration of neoadjuvant treatment options for patient with ER+/HER2– disease.

Variation in surgical practice following NACT was seen, with 44 per cent of centres excising the pretreatment tumour footprint following treatment, and 55 per cent undertaking risk-adapted surgery. Despite this, for 36.2 per cent of tumours requiring mastectomy at diagnosis, the procedure was converted to BCS, in keeping with published data suggesting that modern chemotherapeutic regimens can downstage disease^12^. Patients undergoing BCS were significantly more likely to have a pCR than those undergoing mastectomy, suggesting that, as pCR rates increase with improved patient selection and treatment, it is likely that BCS rates will also rise. This study confirms that a proportion of patients with tumours suitable for BCS will elect to undergo mastectomy. This might be because of multifocal/bilateral disease, patient preference or a mutation in a risk predisposition gene (data on this were not available for the cohort). Following BCS, rates of margin involvement were low and comparable with those reported after BCS without NACT, and this did not appear to be related to whether or not treatment of the tumour was downstaged from a planned mastectomy at baseline^13^.

Downstaging of axillary surgery was less commonly seen after NACT, with greater variation in treatment. SLNB before treatment continues to be performed in patients with cN0 disease, despite the known low false-negative rate after NACT. Not all patients with node-positive tumours underwent axillary reassessment after NACT, and 64.9 per cent of patients with node-positive disease at baseline proceeded to axillary dissection after NACT, with almost half (184 patients, 49.5 per cent) having no evidence of axillary disease. Taken together, these data imply that some patients could undergo less extensive axillary surgery following NACT, but are not currently considered for this. More patients could be offered pretreatment nodal marking and targeted axillary dissection, with the options of either being treated in the UK’s ongoing trial of axillary surgery after neoadjuvant therapy (ATNEC, https://clinicaltrials.gov/ct2/show/NCT04109079), or receiving axillary radiotherapy.

There are limitations to this study. Data were collected from only a proportion of around 150 UK breast units; participating units may be those with a high use of NACT, and so these results may not necessarily be more widely generalizable. Additionally, an observational study introduces the possibility of bias, although measures were taken to minimize this. There are some missing data and, to minimize the impact of this, such patients were excluded from the analysis of pCR and surgical decision-making. Finally, data were collected during 2017–2018, and practice may have changed in the intervening period, in particularly with increasing use of platinums in TNBC further increasing pCR rates in this subtype. Furthermore, since inception of the study, guidelines on surgical management of the axilla have been published in the UK, and this may have changed clinical practice^14^.

The NeST study has demonstrated variation in use of and decision-making around NACT across the UK, with surgical downstaging more apparent in the breast than the axilla, and variation according to disease subtype. These findings highlight the need for clear guidelines for decision-making in terms of patient selection and both breast and axillary surgery, to address treatment variation and optimize patient outcomes.

## Supplementary Material

znac131_Supplementary_Data

## Collaborators

NeST Study Research Collaborative: H. Curry, E. Iddles, M. Mahmood, Y. Masannat, J. Schneider, L. Simpson, M. Sidapra (Aberdeen Royal Infirmary, Aberdeen, UK), L. Baker, H. Capitelli-McMahon, M. Hughes (Airedale Hospital, Keighley, UK), A. Isaac, B. Skelly (Belfast City Hospital, Belfast, UK), C. Sirianni (Betsi Cadwaladr University Health Board, Bangor, UK), N. Hirst, R. Linforth (Bradford Teaching Hospitals NHS Foundation Trust, Bradford, UK), A. Botes, T. Robinson, T. Schrire (Southmead Hospital, Bristol, UK), J. Alfred, H. Lennon (St Helens Hospital, St Helens, UK), D. Dumitru, E. Kleidi (Addenbrookes Hospitasl, Cambridge, UK), F. Hoar (City Hospital, Birmingham, UK), E. MacInnes (Doncaster Royal Infirmary, Doncaster, UK), K. Sharma (Glasgow Royal Infirmary, Glasgow, UK), T. Alaguthurai (Guy's Hospital, London, UK), N. Chand, C. A. Farulla, A. Hayward, B. Pearce, M. Tatterton, S. Laws (Royal Hampshire County Hospital, Winchester, UK), J. Iqbal, M. S. Mirza, K. V. Sainarayanan (Heart of England NHS Trust, Birmingham, UK), L. Humphreys, S. Tayeh (Homerton Hospital, London, UK), S. Jones (St James's Hospital, Leeds, UK), A. Ansari, R. Bate, B. C. J. Wei, B. Gurung, F. M. T. Leone, C. Mitchell, G. Mondani, S. Pilgrim, T. Sun (Glenfield Hospital, Leicester, UK), G. Boundouki (Wythenshawe Hospital, Manchester, UK), R. Broadbent, A. Khan, F. Morgans-Slader (Christie Hospital, Manchester, UK), J. Rai, R. Soulsby (Milton Keynes University Hospital, Milton Keynes, UK), H. Cain, R. Thomas (Royal Victoria Infirmary, Newcastle, UK), B. Elsberger (Ninewells Hospital, Dundee, UK), G. Walls (Altnagelvin Hospital, Londonderry, UK), S. Cadwell-Sneath, J. Couch, M. D’Auria, C. Grundy, S. Hitchin, H. Khout, F. Latief, J. Mondani, A. Nessa, G. Oni, L. Sawers, S. S. Rajan, Q. Tan, L. Whisker (Notthingham University Hospitals, Nottingham, UK), A. Ghoneima, M. Rezacova, N. Marikakis (Queen Alexandra Hospital, Portsmouth, UK), L. Ballance (Royal Preston Hospital, Preston, UK), U. Andaleeb, N. Basu (Queen Elizabeth Hospitals, Birmingham, UK), T. Hubbard, A. Maxwell, M. Roland (Royal Devon & Exeter Hospital, Exeter, UK), C. Weerasinghe (Royal Liverpool Hospitals, Liverpool, UK), Q. Ain, G. Bitsakou, C. Chamberlain, N. Chopra, A. Micha, C. Norman, P. Padmanabhan, N. Patani, K. Shanthakunalan, E. St John (Royal Marsden Hospital, Sutton, UK), S. Jafferbhoy (University Hospital North Midlands, Stoke-on-Trent, UK), C. Bransgrove, A. Hussein, J. Livingstone, O. Waker (Royal Surrey County Hospital, Guildford, UK), J. Hack (Salisbury District Hospital, Salisbury, UK), S. Hadad (Royal Halamshire Hospital, Sheffield, UK), J. Newell (Ulster Hospital, Dundonald, UK), A. Heetun (University Hospital Southampton, Southampton, UK), A. Hargreaves (Warrington and Halton NHS Trust, Warrington, UK), E. Rahman, R. Vidya (Royal Wolverhampton Hospital, Wolverhampton, UK).
