# Corrigendum to: Current practice and surgical outcomes of neoadjuvant chemotherapy for early breast cancer: UK NeST study

**DOI:** 10.1093/bjs/znac282

**Published:** 2022-09-09

**Authors:** Hiba Fatayer, Rachel L O'Connell, Finian Bannon, Charlotte E Coles, Ellen Copson, Ramsey I Cutress, Rajiv V Dave, Matthew D Gardiner, Margaret Grayson, Christopher Holcombe, Sheeba Irshad, Gareth W Irwin, Ciara O'Brien, Carlo Palmieri, Abeer M Shaaban, Nisha Sharma, Jagdeep K Singh, Ian Whitehead, Shelley Potter, Stuart A McIntosh, NeST Study Research Collaborative

https://doi.org/10.1093/bjs/znac131

In the originally published version of this manuscript, the name of the fourth collaborator in the NeST Study Research Collaborative was inadvertently misspelled as Y. Massanat. The correct name is Y. Masannat.

This error has been corrected.

