# Supplementary material for: Current practice and surgical outcomes of neoadjuvant chemotherapy for early breast cancer: UK NeST study
Source: Br J Surg. 2022 May 11;109(9):800–3. doi: 10.1093/bjs/znac131 (PMC10364769; doi:10.1093/bjs/znac131)
Supplement: znac131_Supplementary_Data [file znac131_supplementary_data.docx]

Supplementary material

Methods

All breast surgical units managing breast cancer with NACT were invited to participate through the UK Mammary Fold Breast Trainees’ Association, the Association of Surgeons in Training, the National Trainee Research Collaborative, the Breast Cancer Trainees’ Research Collaborative Group, the NCRI Breast Research Group, the Reconstructive Surgery Trials Network and the Association of Breast Surgery. Consecutive women undergoing NACT as their primary treatment for breast cancer (including chemotherapy, endocrine therapy and targeted therapies) with curative intent at participating centres between 1^st^ December 2017 and 30^th^ November 2018 were recruited to the study. Patients over 16 years with histologically confirmed diagnosis of breast cancer and a multi-disciplinary team (MDT) meeting recommendation of NACT were included. Patients entered into “window of opportunity” clinical trials were excluded. Individual patient consent was not required as this study was classified as service evaluation by the UK National Health Service Research Authority Decision Tool (<http://www.hra-decisiontools.org.uk/research/index.html>). Institutional approval was obtained from each participating institution prior to commencing patient recruitment. Study data were collected and managed using REDCap electronic data capture tools hosted at the Kennedy Institute of Rheumatology, University of Oxford ([30](#_ENREF_30), [31](#_ENREF_31)).

Patients were identified prospectively from outpatient clinics and MDT meetings. MDTs were asked to prospectively record what planned surgery would take place should the patient not have NACT, to capture whether or not patients were eligible for breast conservation at baseline. For patients in whom NACT was recommended, data were collected on whether patients accepted or declined the offered treatment.

**Statistical analysis**

Descriptive summary statistics were calculated for each variable to describe the overall practice and surgical outcomes following neoadjuvant chemotherapy. Counts and percentages were used to describe categorical data and median and inter quartile range (IQR) range to describe continuous data. The protocol did not specify any pre-planned statistical analysis; however, exploratory statistical analysis was carried out using Graphpad Prism v9.0.0. The χ^2^ test was used to compare categorical variables. All comparisons were two sided and a p value of ≤0.05 was considered significant.

## 1091 patients were recommended NST of whom 1020 (93.5%) accepted. A total of 47 patients were recommended NACT by the treating MDT but declined treatment. Of these 26 patients wanted to proceed directly to surgery, five were found to have medical contraindications to NACT following clinical assessment, three patients did not wish to receive chemotherapy as part of their treatment, one patient was offered alternative NACT, and in one patient it was felt that neoadjuvant treatment was unlikely to change the surgical plan. The reason for declining NACT was unknown in two patients.

Table S1: Demographics of patients treated with neoadjuvant chemotherapy. Values in parenthesis are percentages unless indicated otherwise; values are median (IQR)(range). Human epithelial growth factor 2 (HER2), oestrogen receptor (ER), progesterone receptor (PR). Axillary nodal status was determined by axillary ultrasound and either fine needle aspiration (FNA) or core biopsy of any abnormal nodes visualised according to local practice.

|  | **No. of patients (n= 900)**  **N (%)** |
| --- | --- |
| **Age (years)***  **0-44**  **45-54**  **55-64**  **65-74**  **>74**  **Menopausal status**  **Premenopausal**  **Perimenopausal**  **Postmenopausal**  **Unknown**  **Performance status**  **0**  **1**  **2**  **3-4**  **Unknown**  **Clinical T stage (n = 916)**  **T1**  **T2**  **T3**  **T4**  **Unknown**  **Clinical N stage (n=916)**  **Node negative**  **Node positive**  **Unknown**  **Imaging at diagnosis**  **Mammogram**  **USS**  **MRI**  **Other**  **Tumour focality (n=916)**  **Unifocal**  **Multifocal/Multicentric**  **Unknown**  **Presentation**  **Screen detected**  **Symptomatic**  **Unknown**  **Inflammatory breast cancer**  **Tumour characteristics (on pre-treatment core biopsy, n=916)**  **Invasive tumour histological type**  **No special type (NST)**  **Lobular**  **Other**  **Unknown**  **Tumour grade (n=916)**  **1**  **2**  **3**  **Unknown**  **Phenotype**  **HER2 +ve**  **HER2 +ve ER +ve**  **HER2 +ve ER -ve**  **HER2 +ve ER status unknown**  **ER -ve HER2-ve**  **ER -ve PgR -ve HER2 -ve**  **ER -ve PgR +ve HER2 -ve**  **ER -ve PgR U/K HER2 -ve**  **ER +ve HER2 -ve**  **ER+ve PgR+ve**  **ER+ve PgR-ve**  **ER+ve PgR unknown** | 262 (29.1)  297 (33)  217 (24.1)  108 (12)  16 (1.8)  386 (42.9)  70 (7.8)  392 (43.6)  52 (5.7)  821 (91.2)  65 (7.2)  8 (0.9)  0  6 (0.7)  97 (10.6)  565 (61.7)  181 (19.8)  62 (6.7)  11 (1.2)  449 (49.0)  461 (50.3)  6 (0.7)  875 (95.5)  885 (96.6)  469 (51.2)  20 (2.2)  668 (72.9)  208 (22.7)  40 (4.4)  141 (15.4)  774 (84.5)  1 (0.1)  62 (6.7)  792 (86.5)  37 (4)  83 (9.1)  4 (0.4)  13 (1.4)  330 (36)  565 (61.7)  8 (0.9)  426 (46.5)  273 (64.1)  151 (35.4)  2 (0.5)  262 (28.6)  233  8  21  228 (24.9)  112 (49.1)  54 (23.7)  62 (27.2) |

|  | pCR | |
| --- | --- | --- |
| *Pre-treatment pathology* HER2 +HER2+/ER+HER2+/ER-TNBCER+ HER2 - | ypT0, N0148 (34.7)80 (29.3)67 (44.3)81 (30.9)19 (8.3) | ypT0/ypTis, N0205 (48.1)115 (42.1)89 (58.9)101 (38.5)24 (10.5) |
| Total | 248 (27.1) | 330 (36.1) |

**Table S2: Overall pathological response rates in breast and axilla in patients who were node positive at diagnosis (total n=448), presented according to disease subtype (ypT0/ypTis, ypN0).**

## Table S3: Axillary surgery performed following chemotherapy, classified by clinical nodal status at baseline (cN0/cN+) and according to final histopathological nodal status after chemotherapy (ypN0/ypN+).

|  | Final histology | | | |
| --- | --- | --- | --- | --- |
|  | ypN+ | ypN- | Unknown | Total |
| SLNB after NACT (n=405)  cN0  cN+  unknown | 19 (5.3)  11 (25)  0 | 334 (93.6)  33 (75)  4 (100) | 4 (1.1)  0  0 | 357 (88.1)  44 (10.9)  4 (1) |
| Targeted axillary dissection (n=55)  cN0  cN+ | 2 (8)  5 (1.7) | 23 (92)  24 (80) | 0  1 (3.3) | 25 (45.5)  30 (54.5) |
| Primary ALND (n=385)  cN0  cN+  unknown | 5 (41.7)  184 (49.5) | 7 (58)  178 (47.8)  1 (100) | 0  10 (2.7) | 12 (3.1)  372 (96.6)  1 (0.2) |
| Completion ALND (n=25)  cN0  cN+ | 3 (33.3)  8 (50) | 6 (66.7)  8 (50) | 0  0 | 9 (36)  16 (64) |
| Upfront SLNB (n= 38)  cN0  cN+  Unknown | 0  0  0 | 0  0  0 | 36  2  1 | 36 (94.7)  2 (5.3)  1 (2.6) |

##

**NeST Study Research Collaborative**

Aberdeen Royal Infirmary, UK: Heather Curry, Emma Iddles, Maria Mahmood, Yazan Massanat, James Schneider, Louise Simpson, Misha Sidapra,

Airedale Hospital, UK: Liz Baker, Helen Capitelli-McMahon, Michael Hughes

Belfast City Hospital, UK: Anna Isaac, Brendan Skelly

Betsi Cadwaladr University Health Board, UK: Chiara Sirianni

Bradford teaching Hospitals NHS Trust, UK: Natalie Hirst, Rick Linforth

Southmead Hospital, Bristol: Azel Botes, Tim Robinson, Timothy Schrire

Burney Breast Unit, UK: Joshua Alfred, Hannah Lennon

Addenbrooke’s Hospital Cambridge, UK: Dorin Dumitru, Eleftheria Kleidi

City Hospital Birmingham, UK: Fiona Hoar

Doncaster Royal Infirmary, UK: Emma MacInnes

Glasgow Royal Infirmary, UK: Kavita Sharma

Guy’s Hospital, UK: Thanu Alaguthurai

Royal Hampshire County Hospital, UK: Natalie Chand, Clizia Airo Farulla, Abigail Hayward, Belinda Pearce, Mark Tatterton, Siobhan Laws

Heart of England NHS Trust, UK: Javeria Iqbal, Muhammad Salman Mirza, Keerthana Vellore Sainarayanan, Laura Humphreys

Homerton Hospital, UK: Salim Tayeh

St James Hospital, Leeds, UK: Stacey Jones

University Hospitals of Leicester, UK: Aonghus Ansari, Rosie Bate, Bryan Chew Jun Wei, Binay Gurung, Francesca Maria Teresita Leone, Charlotte Mitchell, Giuseppina Mondani, Simon Pilgrim, Thomas Sun

Wythenshawe Hospital, Manchester University NHS Foundation Trust, UK :George Boundouki

The Christie Hospital NHS Foundation Trust, Manchester, UK: Rachel Broadbent, Adeel Khan, Francesca Morgans-Slader

Milton Keynes University Hospital, UK: Jason Rai, Rachel Soulsby

Royal Victoria Infirmary Newcastle, UK: Henry Cain, Robert Thomas

Ninewells Hospital Dundee, UK: Beatrix Elsberger

North-West Cancer Centre, Altnagelvin Hospital, UK: Gerard Walls

Nottingham University Hospitals, UK: Sarah Cadwell-Sneath, Jennifer Couch, Marta D’Auria, Catherine Grundy, Samantha Hitchin, Hazem Khout, Farris Latief, Jo Mondani, Ashrafun Nessa, Georgette Oni, Lisa Sawers, Sreekumar Sundara Rajan, Quing Tan, Lisa Whisker

Queen Alexandra Hospital Portsmouth, UK: Ahmed Ghoneima, Monika Rezacova, Nikolaos Marikakis

Royal Preston Hospital, UK: Laura Balance

Queen Elizabeth Hospital Birmingham, UK: Uzma Andaleeb, Naren Basu

Royal Devon and Exeter Hospital, UK: Thomas Hubbard, Alice Maxwell, Matthew Roland,

Liverpool University Hospitals Foundation Trust, Liverpool, UK: Chamindri Weerasinghe

Royal Marsden NHS Foundation Trust, UK: Quratul Ain, Georgina Bitsakou, Carla Chamberlain, Neha Chopra, Aikaterini Micha, Carol Norman, Pooja Padmanabhan, Neill Patani, Karthika Shanthakunalan, Edward St John

University Hospital North Midlands, UK: Sadaf Jafferbhoy

Salisbury District Hospital, UK: Catherine Bransgrove, Aliyah Hussein, James Livingstone, Olivia Waker

Salisbury District Hospital, UK: Joanna Hack

Royal Hallamshire Hospital Sheffield, UK: Sirwan Hadad

Ulster Hospital Dundonald, UK: Janet Newell

Southampton University Hospital, UK: Adam Heetun

Warrington and Halton NHS Trust, UK: Anita Hargreaves

Royal Wolverhampton Hospital, UK: Ehsanur Rahman, Raghavan Vidya

**Author contributions:**

SMcI conceived the study. SP developed the REDCap database. SMcI, GI, SP, ROC and IW carried out the pilot study. FB, ROC, CEC, EC, RIC, RVD, MGray, CH, SI, GI, CO’B, CP, AMS, NS, JKS, IW, SP and SMcI contributed to the design of the final study. SMcI and MGard coordinated collaborator recruitment and provided collaborator support. CEC, RC, RIC, CH, SI, CO’B, CP, AMS, NS, JKS and SMcI provided clinical leadership and promoted unit participation and data collection. FB provided statistical support. HF wrote the first draft of the paper, which was revised by SMcI. All authors critically reviewed and approved the paper before submission.

**Ethics approval and consent to participate:** Ethics approval was not required according to the NHS Health Research Authority online decision tool (<http://www.hra-decisiontools.org.uk/research/>)

**Competing interests:** CEC is funded by the NIHR and supported by the NIHR Cambridge BRC. SP is an NIHR Clinician Scientist (CS-2016-16-019) and is supported by the NIHR Bristol BRC. EC declares honoraria from Roche, Pfizer, Astra Zeneca, Lilly, Nanostring and expert panel work for the World Cancer Fund. SMcI declares honoraria from Roche, Lilly, Daichii-Sankyo and BARD, institutional research funding from Novartis, and grant funding from the NIHR. RIC utilises equipment to analyse body composition for research purposes provided by Seca to University Hospitals Southampton as part of an NIHR model industry collaborative agreement, and holds research grants from the NIHR and WCRF. AMS is funded by the Birmingham Cancer Research UK Centre (C17422/A25154) and declares advisory board work for Exact Sciences and Veracyte, and an educational grant from Roche. The other authors declare no competing interests.

**Data availability:** Data supporting the results reported in the paper cannot be found on a publicly available database. The data have been uploaded by collaborators to a REDCap database, and individual units have access to their own data, but not the overall national data.

**Acknowledgements:**

The following contributed patients/provided overall supervision at individual hospitals:

Belfast City Hospital: Samantha Sloan, Peter Mallon, Ciara McGoldrick, Jane Hurwitz, Alison Clayton

Bradford Teaching Hospitals NHS Trust: Cathy Tait, M Salhab

Southmead Hospital, Bristol, UK: Jeremy Braybrooke, Charles Comins, Amit Bahl, Vivek Mohan, Jess Jenkins, Zenon Rayter, Rachel Ainsworth, Sasi Govindarajulu, Ajay Sahu, James Cook, Simon Cawthorne

Burney Breast Unit, UK: Leena Chagla, Tamara Kiernan, Atanu Ray

Addenbrooke’s Hospital, Cambridge, UK: John R Benson, Parto Forouhi, Amit Agrawal

Royal Hampshire County Hospital, UK: Kevin Harris, Anne Stebbing, Dick Rainsbury, Sanjay Ray, Umesh Hombaiah

University Hospitals Leicester, Leicester, UK: Jaroslaw Krupa, Calliope Vallassiadu, Sheila Shokoui, Kelly Lambert

Royal Victoria Hospital, Newcastle, UK: Adam Critchley, Loraine Kalra, Nic Cresti, Mark Verrill, Walid Sasi, Dimitrios Dragoumis, Frances Kenny

Wythenshawe Hospital, Manchester University NHS Foundation Trust, UK: Anne Armstrong, Sacha Howell, Andrew Wardley, Cliona Kirwan, Nicola Barnes, James Harvey, Ashu Gandhi, John Murphy, Amanda Taylor, Marina Karina, Hany El’deeb

North-West Cancer Centre, Altnagelvin Hospital, UK: Lucy Jellett, Rhun Evans, Linda McLaughlin, Padraig Diggin

Ipswich Hospital, Ipswich, UK: Caroline Archer

Royal Preston Hospital, UK: Gary Boland, Bade Murthy, Zuhair Saidan

Queen Elizabeth Hospital Birmingham, UK: Daniel Rea

Royal Devon and Exeter Hospital, UK: Alice Maxwell, Douglas Ferguson, Sisse Olsen, Rachel Tillet, Charlotte Ives, Julie Dunn, Kate Scratchard, Jennifer Forrest, David Wang, Chris Hamilton, Andrew Goodman, Ann McCormack, Peter Stephens

Royal Marsden NHS Foundation Trust, UK: Jennifer Rusby, Peter A Barry, Katherine Krupa, William Allum, Fiona McNeill, Nicola Roche, Gerald Gui, Mario Konstantinos Tasoulis, Marina Parton, Alistair Ring, Nicholas Turner, Alicia Okines, Sophie McGrath

Royal Surrey County Hospital, UK: Tracey Irvine, Polly Partlett, Elizabeth Clayton, Farokh Parzad, Jonathan Horsnell, Tim Crook, Anthony Neal

Ulster Hospital, Dundonald, UK: Lynn Darragh

Warrington and Halton NHS Trust, UK: Joanne Cliff, Ansar Farook, Noaman Sarfraz, Azher Shafiq

## This study was supported a research development grant from the Association of Breast Surgery. The NeST Study Collaborative are grateful for the support of the Reconstructive Surgery Trials Network.
